# Retrospective analysis of the development history of the Chinese food additive standards system based on the CODEX principles

**DOI:** 10.1038/s41538-019-0060-x

**Published:** 2019-12-16

**Authors:** Zhongdong Liu, Boxiang Liu, Gaowei Chen

**Affiliations:** 1 Henan University of Technology, Henan, China; 2 Department of Biology, Stanford University, Stanford, CA 94305 USA; 3 Baidu Research, Sunnyvale, CA 94089 USA

**Keywords:** Quality of life, Nutrition

## Abstract

In 1984, China joined the Codex Alimentarius Commission (CAC), which was established by the United Nations’ World Health Organization (WHO) and the Food and Agriculture Organization (FAO), which consists of 188 member states and one member organization. Since then, China has taken an active role in various initiatives organized by the Codex Committee on Food Additives (CCFA) and has shared resources and experience with its Codex member states, thus effectively promoting the development of the Chinese food additive standards system. Instead of a country where almost no systematic food additive standard were available, China has become the host country of the CCFA’s sessions. China’s food additive industry is the only one that is supported by international standards, out of the industries of the International Standard Industrial Classification and China’s national economy. Based on this case, four strategic milestones are summarized by retrospectively analyzing the history of the development of Chinese food additive standards from 1978 to the present. China is expected to share its valuable experience and provide references for the improvement of food additive standards systems in multiple developing countries, so promoting food safety and trade harmonization. With the advances in core technologies in the food industry, the future development of food additive standardization is also forecast in this review.

## Preface

Over the past 40 years, Chinese food additive standards system have undergone tremendous development. Zhang et al have published detailed description of Chinese food safety legislation process.^1^ However, this paper reviews the different development stages of China’s food additive standards system and summarizes the problems encountered in various stages of China and the solutions to overcome them.

**Fig. 1:**
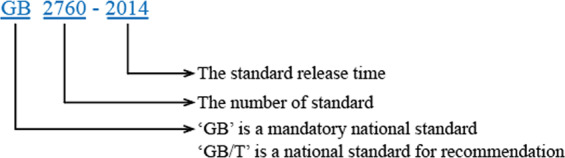
Symbols of the Chinese National Standards.

China had almost no formal concept of food additives before 1978. Its food production was mainly based on traditional methods and performed manually or with simple equipment.^2^ Food additives used in those years mainly included yeast, baking soda, monosodium glutamate, artificial sweeteners and food colors. Only two official documents issued by the Ministry of Health were available during those years, namely, the Regulation on the Dosage of Sweetener in Foods issued in 1954 and the Hygienic Standard for Uses of Food Additives (GBn 50–77) (meaning of symbols as shown in Fig. 1) in 1977. Fifty seven of food additives were merely listed in these documents, which did not specify usage or limits. Consequently, food additives were insufficiently regulated.

Since the economic reform in 1978, China’s food industry has experienced historic changes, which are mainly shown in three characteristics, namely: (1) the productions of major foods (e.g., cereals, fats, meats, eggs, dairy, sugar, fruits, vegetables and aquatic products) has experienced dramatic increases.^3^ For example, the yield of cereals has increased to 618 million tons in 2017 from 300 million tons in 1980; (2) the food rationing system was discontinued. For instance, staple and non-staple food coupons (for cereals, fats, meats and sugar) in place since 1955 were abandoned in 1993. This signaled the end of China’s food subsistence problems and was followed by a constant reduction in the universal Engel’s coefficient, and; (3) the Chinese food industry was open to the world, including its huge domestic market with 1.4 billion population, among whom 300 million people are at the middle income level. It is indicated in the Report on the Development of Chinese Residents’ Consumption in 2017^4^ prepared by the National Development and Reform Commission that the total China Engel coefficient in 2017 is 29.39% and China is rated by the UN into the welfare range of 20–30%. With the development of the food processing industry, relevant laws and regulations on food additives were gradually formulated and improved by reference to imported products and the experience of other countries. Therefore, it is believed that the Chinese food additive standards system developing together with China’s food industry is one of the earliest and most complete Chinese standards and specifications systems, which are in line with international trends and in close relation to the daily life of Chinese residents.

Based on the statistics from the National Health Commission of the People’s Republic of China (NHC), until July 11 of 2017, out of the 1224 national standards for food safety, there were 586 standards for the quality, specifications and other matters of food additives, among which 29 were standards^5^ for the quality and specifications of food nutrition supplements. Until 2017, there have been 2362 types of approved and notified food additives. All these have become an important part of major technical specifications and food safety assurance system of China’s food industry and represent a successful shift to the modern dietary structure of Chinese citizens who now constitute 1/5 of the global population.

According to above analysis, it is concluded that China has completed four strategic stages, which include critical factors and characteristics, based on the WHO/FAO’s food safety risk management framework and Codex principles. The factual evidence and analysis are as follows.

## Reference in the stage of “Startup of Standards”

Since 1979, China has fully imported food additives into food processing and preserving by following the foreign best practices. In 1981, the national mandatory standards for food additives laid the foundation and as a result The Hygienic Standard for Uses of Food Additives (GB2760-81)^6^ was promulgated, which outlined 16 categories and 64 food additives, which was expanded to 2468 food additives in 2014 (GB2760-2014). The problems after opening trade in food were how to satisfy the needs for management and application of thousands of food additives directly and indirectly imported into China, and how to quickly establish the food additive standards system accordingly. The decision makers at that time made strategic choices, namely: (1) referring to the complete, effective, authoritative and scientific global standards; and (2) primarily referring to the Codex system of CCFA and CCFA members. The detailed analysis is given in Section “Analysis on the reference to the standard system of CCFA and its members”.

### Analysis on the reference to the standard system of CCFA and its members

The CCFA system provides significant shared resources of food additive standards from many developed countries. China has referred to the quality specifications and standards for food additives with 21 categories and more than 1600 food additives from CCFA and its members. The detailed analyses are given in Table 1.

**Table 1:** Research on the factors with reference to international standards.

Chinese standards system, categories and products	Standards of the Codex member countries referred to by China	Reasons, basic contents and number of reference(s) made to by China	Critical indicators referred to by China	Current status of standards of China and Codex member countries
Food additive standards system (GB2760)	Standard framework, product categories, standard contents, and retrieval methods of CODEX STAN 192^6^	1005 Codex food additive standards and 41 hygiene or technical specifications	Principles of the Codex stan 192–1995 and all indicators related to food safety	China became the leader of CCFA in 2007.
Nutritional supplements standard system (GB14880)	Codex’s dietary supplements (called ‘Nutrition Supplements’ in China)	45 standards of Codex dietary supplements	Intake indices	Separated standards system of food additives and nutrition supplements in China
Consumer Protection Act, and regulations on fair and just trade	11 resolutions, guidelines and documents of Codex’s commerce committee	China became the formal member of CAC in 1984.	WTO (as the General Agreement on Tariffs and Trade), all the relevant CCFA information on maintaining fair trade	Nationwide communication and implementation of food additive standards and regulations to avoid the trade argument caused by the standard difference of food additives
Flavor enhancers in GB2760 with 7 product specification standards	Japan’s Specifications and Standards for Food Additives^16^	Food culture system of neighboring countries and the same East Asian region	Sensory, contents, heavy metals	China and Japan hold seminars at least twice a year
Food colors in GB2760 with 40 product specifications standards	Japan’s Specifications and Standards for Food Additives	Processing industry with the same resources and processing methods; shared products of natural colors	Type, contents, heavy metals	Both countries have jointly applied for Codex international standards for food colors
Emulsifiers in GB2760 with 20 product specification standards	Japan’s Specifications and Standards for Food Additives	Application development of Japanese products in China market	Contents, heavy metals, trans-fatty acids (TFA)	Aligned emulsifier standards of both countries
Preservatives in GB2760 with 7 product specification standards	Related terms in CFR and FDA in US^17^	Rigorous fundamental research on scientificity and safety	Latest medical research results, contents, heavy metals	China and US hold the world’s largest product seminar conference twice a year.
Flavors and fragrances, with the list of 940 products	FEMA^18^	Adopted by 110 counties in the world	Type, contents, heavy metals	Establishing the strategic partner relationship with FEMA
Nutrition supplements in GB14880 with 20 product specification standards	Related terms in CFR and FDA in US	Innovative products, closely related to nutrition and feed standards	Normalization principle, contents, heavy metals and TFA	Joint extension of dietary supplementary applications by both countries
Enzymes in GB 2760 with 17 product specification standards	EU Standards^19^	Regulation and policies related to fermentation are with longer history.	Application scope and dosage in meat, dairy and baked products	Reference of application of starter culture and enzyme in non-Chinese traditional foods
Thickeners in GB2760 with 10 product specification standards	EU standard, carry-over rules and principles	Product bases in multiple countries, especially in Africa and Asia	Type, contents, heavy metals, and toxicological evaluation	Reference on the application standard of food additives in organic foods
Sweeteners in GB2760 with 20 product specification standards	EU standards	Origins of manufacture technology of chemical products, with traceability under management regulations	Type, sweetness and substitution of sugar	Health guidance of the sugar dosage in foods
Standards of gum base and its ingredients in gum based candy (GB 2760)	EU standards	Management regulation for edible chemicals in non-Chinese traditional foods	Safety standards for foods majorly with food additives	Extension of food additive field towards food ingredients

By systematically referring to international general standards, China had filled the big gap in the formulation, management and use of food additive standards in five years. Based on GB2760-81, The Hygienic Standard for Uses of Food Additives (GB2760-86)^7^ was promulgated in 1986 to cover 21 categories of food additives (625 food additives in total) commonly used in the food processing industry. This strategy had the following major effects: (1) meeting the urgent needs for China’s food processing industry to shift from traditional to modern food processing; (2) building the regulatory framework for risk assessment and management and technical specifications for food additives in China; (3) having access to a method to quickly improve standardization technical capability through an international network. Within the next ten years, the second improvement strategy was implemented in China by referring to Codex standards, with detailed analysis as follows.

### Improvement of the Chinese food additive standards system through Codex

The Standardization Law of the People’s Republic of China was approved on Dec. 29, 1988.

**Fig. 2:**
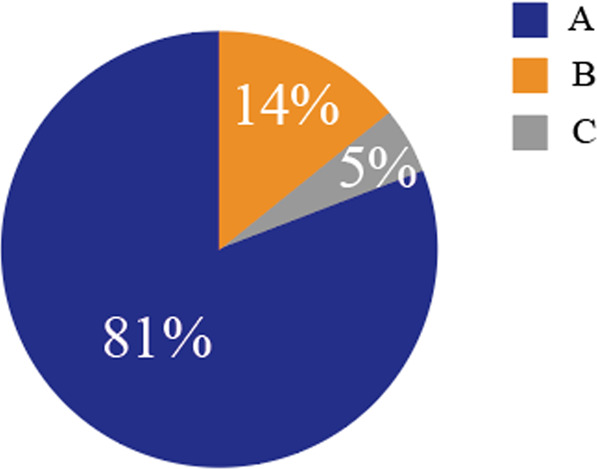
Comparison analysis of the ratio of standards drafted based on three methods. (A) Chinese standards formulated by Codex and related standard data; (B) Chinese standards developed in cooperation with Codex Member States; (C) Independent Chinese standards.

Modern production in China was under regulatory administration of GB2760-86, which has become one of programmed regulations for China’s modern food industry. In order to fully implement the effective management and control policies, the Chinese food additive standards were improved as a critical strategy, with the characteristics described below: (1) a wide range of products were involved and more than 900 local, industry and national standards were revised; and (2) active participation in Codex activities was undertaken, such as presence at CCFA meetings, seminars on Hazard Analysis and Critical Control Point (HACCP), risk classification and genetically modified organisms (GMOs), organizing the 9th CCASIA (1994) and the co-hosting of the 34^th^ CCFAC (2000), etc. The following three methods for the improvement of the Chinese food additive standards system: (1) the development and review of the Chinese standards are based on the extensive reference to Codex and related standards and data. For example, it is clearly specified that the standard data and documents of at least two CCFA member states and organizations should be enclosed in any application for development/revision of Chinese standards; (2) working with Codex member states to develop standards, and; (3) independently developing standards for such products with Chinese characteristics only. Figure 2 shows the number and ratio of standards developed based on such three methods. Base and its ingredients in gum based candy (GB 2760) regulation for edible chemicals in non-Chinese traditional foods foods majorly with food additives additive field towards food ingredients.

**Fig. 3:**
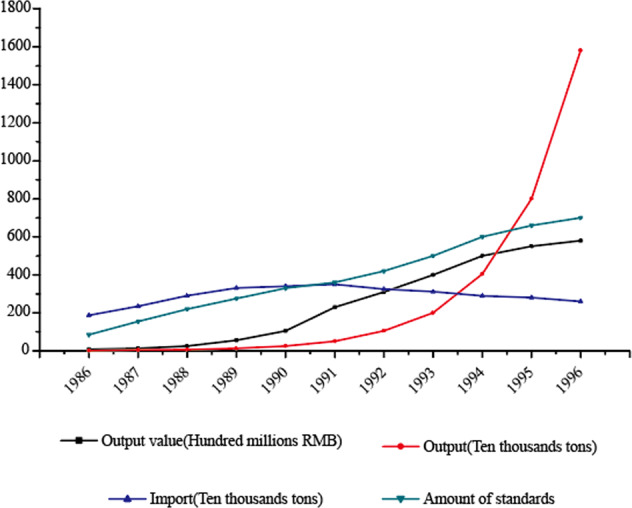
Progress analysis of the Chinese food additive import and output.

Even though there were limited standards drafted by China independently, the standards system has sufficiently improved and has promoted the China’s food additive industry from importing to a manufacturing leader. See Fig. 3 for details.

During this period, an average number of about 60 standards were promulgated every year and a relatively complete industrial technical specifications for food additives were established in China within ten years. Subsequently, the Chinese food additive standards stepped into the modernization development period, which is the 3^rd^ strategic process.

## Modernization of Chinese food additive standards

Both strategic processes above, as well as the publication of GB2760-1996 (including 1240 food additives) signaled the transition of the basic food additive standards system in China from the basic to the substantial level. The subsequent challenge to China was how to realize the modernized within a reasonable and sustainable process. The key factors of this strategy are: (1) close cooperation with development needs of the food industry as one of China’s pillar industries; (2) fulfill the standard revision with the Chinese characteristics in a scientific and reasonable manner; and (3) international harmonization of Chinese food additive standards. The typical three characteristics are analyzed in Sections “To meet the modern management needs of China’s food industry”–“Modernization of the Chinese food additive standards“.

### To meet the modern management needs of China’s food industry

The licensing system is applied in the management of China’s food industry. How can the mandatory requirements for food additive be met quickly during review for permission? In the characteristic Chinese practices, ‘equal adoption’ prevails. The government directly designates complete and general standards from Codex or its member states as the Chinese food additive standards. In this way, millions of ‘equally adopted’ Codex member’s standards are designated, such as those for 20 types of ester-based products (e.g., geranyl formate) used in flavors and 21 types of modified starches as quality improvers represented by hydroxypropyl starch. These standards have accelerated the development of China’s food industry as a pillar industry and improved the standardization system. (In 2017, the food industry output was up to RMB 10.7 trillion/USD 1.7 trillion, and the food service output was up to RMB 4.0 trillion/USD 0.6 trillion).

### Rationalized amendment

Both China and the Codex face challenges of adapting to the dietary traditions under the political and regulatory mechanisms of different countries. Starting with exact duplication of standards, more attention has been paid to the structural characteristics of the Chinese foods in consideration of the Codex standards during the promulgation of standards. For example, the best sensory performance of Chinese meals needs the supplement of main meal, which is steamed or boiled, in white or light color, with light or non-baked flavor. This requires the amendment on the quality enhancers for main meal products, such as the Chinese steamed bread and rice. For instance, dibenzoyl peroxide is not allowed for use in wheat flours; likewise, the characteristics of Chinese liquors are fully considered in allowing for the use of various flavors in Chinese liquors. In addition, the characteristics of the Chinese soy source are also taken into account in the amendment to the food additive standards for seasonings to allow the use of caramel color.

### Modernization of the Chinese food additive standards

**Fig. 4:**
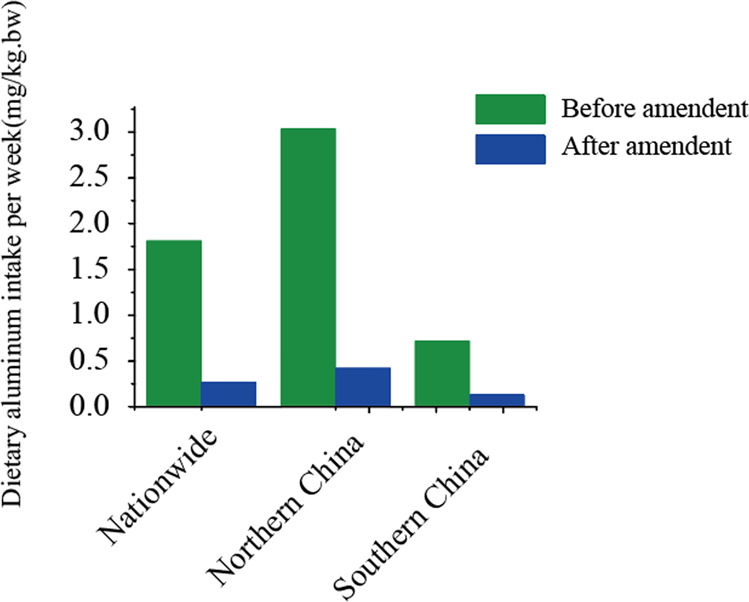
Test results of the China National Center for Food Safety Risk Assessment (CFSA), available 4 months after terminating the use of aluminum-contained raising agents in the Chinese staple foods, such as steamed breads.

The modernization of the Chinese food additive standards represents three approaches: (1) modification of standards from other countries, like US; (2) adoption of concepts, like those in European Union regulations; and (3) production standards based on those from such countries as Japan and South Korea. For example, based on the results of the study of dietary nutrition of consumers in China^8^ and the latest research results from the US on the risk assessment of elemental aluminum,^9^ with the adoption of ‘Directive’ format of EU regulations, the aluminum-contained chemical raising agent is replaced with food additives produced by biological fermentation, which were developed in Japan. Thus the exposure to elemental aluminum in the Chinese staple foods^10^ has been reduced and demonstrates the effects of the management and control of food additives^11^ (as shown in Fig. 4).

Using the three approaches above, the strategic objective of the Chinese standards modernization has shifted to ‘rationalization’ from the initial ‘duplication’. Consequently, Chinese standards are often accepted by the Codex and play a key role in the modern international standards system.

## Constructive development of the Codex and Chinese standards

China has become the host of the Codex Food Additive Committee in 2007 and has itself aligned it standards with those of Codes. For example, GB 2760-2007^12^ states that ‘this standard adopts the format and structure of CODEX STAN 192…’. GB 2760-2007 basically follows the Codex in additive listing, searching, and application methods and attachments, etc. As a result, other countries of the world can refer to and review the format of the standard effectively and efficiently. Since then, the Chinese food additive standards have become a member of the *Union of Global Standards*, which facilitates international trade as well; at the same pace as countries with comparable legal systems, the Chinese national strategy of legal construction of standards has been implemented successfully. The positive evidence is presented and analyzed in Sections “Assurance of legalization policy and strategy of China”–“Innovation of China in the development of its food additive standards”.

### Assurance of legalization policy and strategy of China

China’s food modernization urgently needed legal construction since food safety incidents in China significantly affected the confidence of the public at home and abroad. These incident were very costly and great lessons were learned from them. The Food Hygiene Law of the People’s Republic of China^12^ was promulgated in 1995, and the Food Safety Law of the People’s Republic of China^13^ was issued in February 2009 and amended in 2015. In the food industry, administrative rights are executed in accordance with these laws, and GB 2760 is implemented as common effective rules. The current applicable Food Safety Law (Edition 2015) has 101 mentions of the key words of ‘food additive’. The food additive standard contains the bases for the establishment of regulatory principles and stringent management.^14^

### To prevent the standard from being manipulated by any special interest group

As the Codex global standards, any amendment to such standard could trigger global implications. Such standards could be influence by certain groups because there is usually a range and flexibility in scientific interpretation of experimental results and selection of safety factors. An enterprise could benefit from the establishment of standards that favors their product. For example, a metabolite generated by aspartame (i.e., phenylalanine) can be a potential risk to people with phenylketonuria (PKU), which has been minimized by some entities. Therefore, China maintains alignment with the Codex principle that development of any standards should include: (1) fairness and justice; (2) amendments be based on scientific research results or more emphasis on consumer protection, and: (3) always be based on the risk management structure and scientific experiment outcomes to prevent any groups with interests from advocating standard amendments and terms for commercial purposes only.

### Innovation of China in the development of its food additive standards

The innovative thinking of China on such standards appears strategic in consideration of global needs and focuses on actual innovations. For example, an individual food additive product is normally approved for a single technical effect, which may not satisfy the actual needs for the food quality where a blend of two or more than two food additives give better effects. CCFA’s discussion topics on the ingredients related to composition are not addressed. Moreover, there was progress on the secondary ingredients between 2008 and 2010, which has become the bottleneck for the efficient application of thousands of food additives. In such context, China became the first to develop relevant standards in 2005 (earlier than the EU and Australia) and promulgated the National Standards for Food Safety–General Principles of Composed Food Additives (GB 26687-2011).^15^ Through the Application of Sanitary and Phytosanitary Measures Agreement notifications to the World Trade Organization, China has achieved international recognition and completed the innovation of such standards. After this breakthrough, China discussed secondary food additives for food colors and nutrition supplements in August 2018. China plans to further expand the relevant scope based on this breakthrough and to continue to lead the innovation of international standards of composed food additives and their ingredients.

## Conclusion and prospect

### Conclusion

Over the past forty years, China has become known as a decision-making country by implementing the four strategies above: Initiation (foundation of the standards, recognition of international laws) → improvement (reference to standards, adherence to international laws) → development (standard amendments, absorbing and adoption of international laws) → innovation (contributions to standards, familiarity with international laws). For example, the Chinese programmed food additive standards GB 2760 has evolved over the years: GBn50-77 → GB2760-81 → GB2760-1986 → GB2760-1996 → GB2760-2007 → GB2760-2011 → GB2760-2014 → GB2760-201 × (under amendment). China has completed its food additives standards system consistent with international rules, which not only represents the successful evolution of the food additive standards, but also the vital development of food industry. More importantly, this progress is synchronous with the success of China alleviating poverty and rejuvenating the national economy. Therefore, this example may suggest a means to reduce poverty of developing countries. Apart from the resources and diligence, the success of the Chinese model indicates that it is necessary to construct legal systems in the fundamental areas of society, such as foods. In order to complete this institutional revolution (called ‘reform’ in China) and establish general laws and regulations, political strategies as aforesaid four steps should be considered. A standard and regulation system should be built with specific domestic characteristics with adaption and harmonization of current modern international rules. Practices of China prove that such legal systems are beneficial to promote fair trade and for the steady development of economy and society.

The global relations among countries operate two systems, namely the political system and the economic system. Both are closely related to the Codex. The regulation of food additives is widely beneficial to society and the people, so the changes are readily accepted by the whole society, including industry. It is expected that the practice of China could be taken as an example and the regulation of an industry may be adopted as a breakthrough, so that progress of developing countries could be promoted and finally be harmonized into the global system of national relationships.

### Prospect

Through the participation of CCFA, the food safety risk management in China is becoming more scientific and effective. China has already achieved capabilities of risk assessment and management. In the future, the status and products related to risk assessment, as well as international standards will be continuously followed up and analyzed. Any insufficiency in food additive standards will be scientifically and carefully handled to facilitate the progress of innovative technologies. For example, 3D-printed cakes, candies and biscuits has been commercialized but there is no application standard for food additives or ingredients for 3D food materials. China could take lead to fill the gaps of risk assessment and food additive standards related to 3D foods, as well as other technological advances that are sure to come.

### Reporting summary

Further information on research design is available in the Nature Research Reporting Summary linked to this article.

## Supplementary information

Reporting Summary

## Data Availability

Data available on request from the authors.
